# Unveiling fungal strategies: Mycoremediation in multi-metal pesticide environment using proteomics

**DOI:** 10.1038/s41598-024-74517-y

**Published:** 2024-10-05

**Authors:** Priyadarshini Dey, Anushree Malik, Dileep Kumar Singh, Sven-Bastiaan Haange, Martin von Bergen, Nico Jehmlich

**Affiliations:** 1 Applied Microbiology Lab, Indian Institute of Technology Delhi, Centre for Rural Development and Technology, Hauz Khas, New Delhi, 110016 India; 2 Department of Zoology, University of Delhi, New Delhi, 110007 India; 3 Department of Molecular Toxicology, Helmholtz-Centre for Environmental Research-UFZ GmbH, 04318 Leipzig, Germany; 4 Institute of Biochemistry, Faculty of Biosciences, Pharmacy and Psychology, University of Leipzig, 04109 Leipzig, Germany; 5 Department of Biotechnology, MS Ramaiah Institute of Technology, MSR Nagar, Bengaluru, 560054 India

**Keywords:** Multimetal, Lindane, Haloalkane dehalogenase, Homogentisate 1,2-dioxygenase, Microbiology, Systems biology, Environmental sciences

## Abstract

Micropollutants, such as heavy metals and pesticides, inhibit microbial growth, threatening ecosystems. Yet, the mechanism behind mycoremediation of the pesticide lindane and multiple metals (Cd, Total Cr, Cu, Ni, Pb, Zn) remains poorly understood. In our study, we investigated cellular responses in *Aspergillus fumigatus* PD-18 using LC-MS/MS, identifying 2190 proteins, 1147 of which were consistently present under both stress conditions. Specifically, Cu-Zn superoxide dismutase and heat shock proteins were up-regulated to counter oxidative stress and protein misfolding. Proteins involved in intracellular trafficking, secretion, and vesicular transport; RNA processing and modification showed enhanced abundance and regulating stress response pathways. Additionally, haloalkane dehalogenase and homogentisate 1,2-dioxygenase played pivotal roles in lindane mineralization. Bioinformatics analysis highlighted enriched pathways such as Glyoxylate and dicarboxylate metabolism and Purine metabolism, that are crucial for combating adverse environments. We identified the hub protein 26 S proteasome regulatory subunit complex as potential biomarker and remedial targets for mycoremediation of wastewater, suggesting practical applications for environmental remediation.

**Supplementary Information:**

The online version contains supplementary material available at 10.1038/s41598-024-74517-y.

## Introduction

Organochlorine pesticides are persistent because of the presence of cyclic resonance structure, polar functional groups in their molecules, lipophilic nature and chlorine-substitution. These features enable them to accumulate in animal tissue and the environment. Even though it is banned the organochlorine pesticide hexachlorocyclohexane (HCH) is used by farmers for agriculture purpose. Consequently, this pesticide seep into the water bodies due to agriculture runoff and pose a significant environmental hazard because of its toxicity and long-lasting presence in the environment^1–4^.

Heavy metals are elements having atomic density more than 4000 kg/m^3^ including copper, cadmium, zinc, chromium, arsenic, boron, cobalt, nickel, mercury and lead etc. Certain heavy metals like copper, zinc, nickel, boron, iron, and molybdenum are essential nutrients for organism growth, but they can be harmful to organisms when present in excessive concentrations beyond permissible limits. Other heavy metals such as lead, mercury, cadmium, and arsenic are not essential for the growth of plants and animals. Soil pollution by heavy metals can occur due to the introduction of industrial wastewater, sewage sludge, fertilizers and the weathering of soil minerals^5–8^. Thus these micropollutants must be remediated in a sustainable manner.

A range of chemical, physical and biological methods have been evaluated and utilized for remediation objectives. Physical remediation methods like adsorption, membrane separation, and gravity separation rely on specialized and costly equipment. Similarly, chemical approaches such as precipitation or the Fenton process necessitate the use of large quantities of expensive chemicals, thereby increasing the overall cost of the remediation process^9^. However, biological approaches utilizing free or immobilized cells and enzymes of algae, bacteria and fungi provide superior conversion rates, enhanced selectivity, and lower energy and capital expenses when contrasted with physico-chemical methods^10–12^. The mycoremediation approach to transform and degrade these recalcitrant pollutants stands out for its superior efficiency when compared to other microorganisms such as bacteria and algae^13^.

This is due to fungi’s prevalence in various environments, possessing efficient enzymatic systems viz. peroxidases, laccases, cytochromes P450, hydrolases, esterases, monooxygenases, dioxygenases, dehydrogenases that possess broad specificity and consequently facilitate transformation of plethora of organic contaminants^14^. Moreover, fungi have significant surface-to-biomass ratio, longer life cycle, and extensive mycelial networks that penetrate soil, along with their ability to detoxify contaminants effectively^15–18^. Besides, the majority of metabolites generated through fungal biotransformation of chlorinated pesticides are non-toxic, unlike the toxic intermediates such as pentachlorocyclohexane, 2,5-dichlorohexane that are produced during bacterial degradation processes^19^. The majority of the pollutant-degrading organisms are classified under the phyla *Ascomycota*^20^ and *Basidiomycota*, with a smaller proportion belonging to the subphylum *Mucoromycotina*^18^.

While scattered literature exists on the proteomic studies of heavy metal accumulation or pesticide degradation by various fungi. In a recent study, *Pycnoporus sanguineus* efficiently degraded 5 mg/L triphenyl phosphate pesticide to 62.8% via methylation, hydroxylation and oxidative cleavage with the help of enzymes methyltransferases, cytochrome P450, aromatic compound dioxygenase, oxidizing species-generating enzymes and MFS general substrate transporters^21^. In another study, increased level of antioxidant enzyme glutathione was upregulated in *Rhodotorula mucilaginosa* under exposure to 100 mg/L of arsenic and cadmium heavy metals^22^. Further, there was notable upregulation of cyanide hydratase in cultures containing alachlor herbicide suggests that this enzyme may be a key player in the biodegradation pathway of 50 mg/L alachlor in the fungus *Paecilomyces marquandii*^23^. There was regulation of translational and post-translational processes, various transport mechanisms, numerous metabolic pathways, and the cytoskeleton in order to overcome the oxidative stress induced by copper exposure in *Phanerochaete chrysosporium*^24^. Yet, fungal ability to withstand multiple metals in presence of pesticide lindane as well as fungal pathway for lindane degradation has not been previously explored.

This unaddressed aspect is studied by proteomics that illuminated the protein abundance in *Aspergillus fumigatus* PD-18 encompassing the translational aspects. Hence, a combination of both stresses, pesticide lindane and multi-metals (Cd, Total Cr, Cu, Ni, Pb, and Zn) at levels (0, 30 mg/L) would give a better understanding into the mechanisms adopted by filamentous fungus to evade these deleterious effects and aid in its potential applications in industrial waste remediation.

## Results

Fungi adapt to environmental stress by making alterations in the structure and function at cellular level^15^. In this study, the total proteome analysis indicated that mycoremediation of multiple toxic metals was inhibited in the presence of the co-contaminant pesticide, lindane. Despite this, viable fungal cells were maintained under these conditions, and the metabolic processes involved in the homeostasis of both the multiple metals and pesticide were investigated. The biotic control consisted of fungal cells cultivated in composite media without the addition of heavy metals or lindane.

Here, we used *Aspergillus fumigatus* PD-18 that has been previously been found to subsist and grow on the contaminated river bank^13,25^. *A. fumigatus* PD-18 accumulated multi-metals Zn (98%), Pb (95%), Cd (63%), Cr (62%), Ni (46%), Cu (37%) from the multi-metal and lindane mixture in composite media amended with 1% glucose. Specifically, the absorption of Pb and Zn increased in the presence of lindane. Notably, lindane was degraded to 94% in 72 h. The utilization of lindane by the fungus as a carbon source was depicted by the higher biomass (5.89 ± 0.03 g/L) obtained at 72 h than the biotic control biomass (5.42 ± 0.01 g/L)^13^.

### Proteome abundance in biotic control, multi-metal + pesticide treated *Aspergillus fumigatus* PD-18

Protein profiling conducted on six samples of fungal isolate *Aspergillus fumigatus* PD-18 subjected to the conditions of biotic control and 30 mg/L multimetal + 30 mg/L lindane exposure and their respective sets of three technical duplicates that led to identification of 2190 proteins confidently, that were upregulated/downregulated across both the conditions. 386 and 657 proteins were exclusively present in pesticide + multi-metal extracts and biotic control, respectively.

**Fig. 1:**
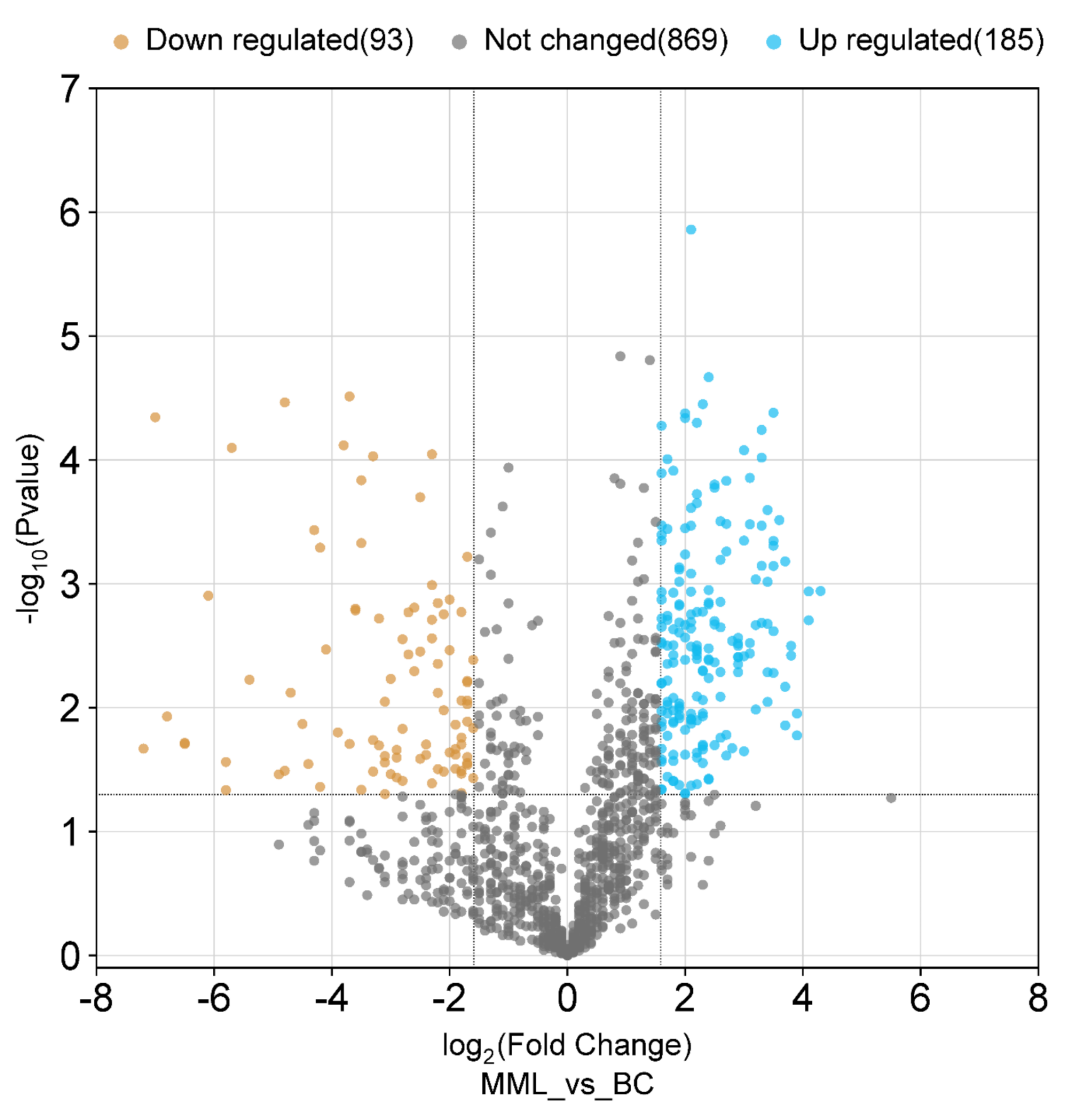
Volcano plot depicting distribution of proteome abundance in *A.fumigatus* PD-18 in presence of 30 mg/L multimetal + 30 mg/L lindane (MML) exposure and proteins without exposure (BC) according to p-value and log2 fold change, indicating significance level at 0.05 and fold change at − 2.0 and 2.0. Blue dots represent upregulated proteins, brown dots represent downregulated proteins, and gray dots represent proteins that were not differentially abundant.

Figure 1 displayed the Volcano plot of significant fold increase and decrease in proteins’ abundance under the exposure of 30 mg/L multimetal + 30 mg/L lindane. Among them, 185 proteins were differentially upregulated while 93 proteins were differentially downregulated and 869 proteins showed no change in regulation. Typically, the upregulated proteins are stimulated to exhibit the fungal resistance against the pollutants’ toxicity, whilst the downregulated proteins are suppressed by the action of the hazardous contaminants.

Few of the proteins that were upregulated by folds were ATP-dependent RNA helicase (4.3), isoamyl alcohol oxidase (4.1), phosphoserine aminotransferase (4.1), aminotransferase (3.9), 14-alpha sterol demethylase (3.9), 26 S protease regulatory subunit (3.8), serine/threonine-protein phosphatase (3.7), proteasome regulatory particle subunit Rpt3 (3.7), eukaryotic translation initiation factor 3 (3.7), glutamyl-tRNA synthetase (3.5), acetyl CoA hydrolase (3.5), glycolipid transfer protein HET-C2 (3.5), oxidoreductase short chain dehydrogenase/reductase (3.5), glutamate dehydrogenase (3.5), mitochondrial inner membrane translocase (3.4), kynurenine aminotransferase (3.4), chitinase (3.3), acetyltransferase (3.2), superoxide dismutase (3.1), hydrolase haloalkane dehalogenase (2.9), acireductone dioxygenase (2.1), homogentisate 1,2-dioxygenase (1.7), respectively. Proteins that were downregulated by folds antifungal protein (− 7.2), 60 S ribosomal protein L37a (− 6.8), endo-chitosanase (− 6.5), hydrophobin (− 6.1), cerevisin (− 5.8), ribotoxin (− 5.8), fucose-specific lectin (− 5.7), methyltransferase (− 5.4-fold).

Thus, highest protein abundance belonged to posttranslational modification, protein turnover, chaperones.

**Fig. 2:**
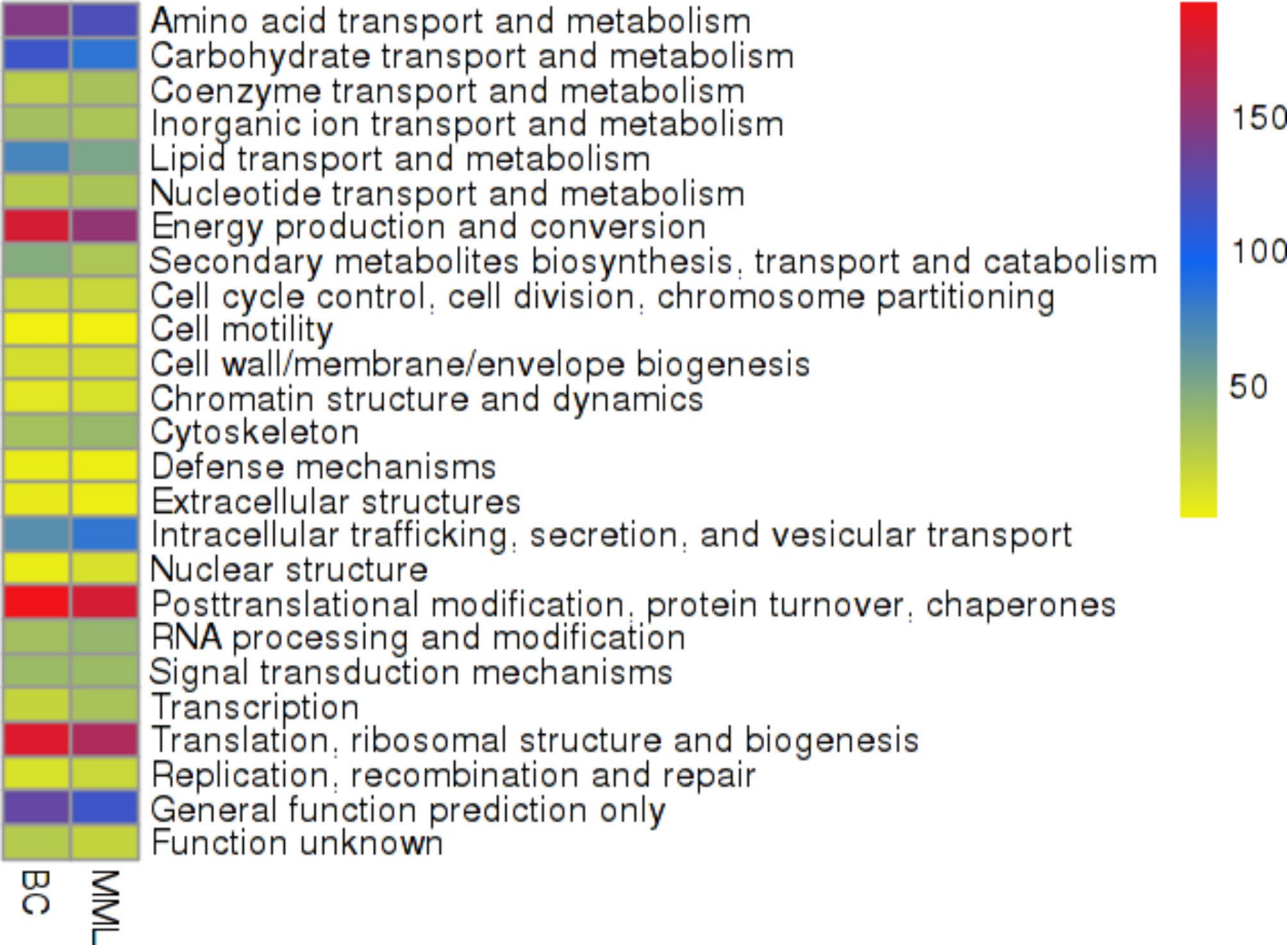
Heatmap visualization of proteome abundance and regulation as red = higher values, dark blue = mid values, yellow = lower values depicted on (x-axis) in *A.fumigatus* PD-18 in presence of 30 mg/L multimetal (MM) + 30 mg/L lindane exposure and proteome abundance without exposure (BC).

In Fig. 2 a heatmap illustrates the differentially regulated proteins of the pesticide + multimetal exposure when contrasted to the biotic control. These were further classified to 23 KOG classes.

Exposure of *A.fumigatus* to multimetals and pesticide lead to significant changes in the proteome abundance. Proteome related to coenzyme transport and metabolism; nucleotide transport and metabolism; cell cycle control and cell division and chromosome partitioning; cell motility; cell wall/membrane/envelope biogenesis; chromatin structure and dynamics; cytoskeleton; intracellular trafficking and secretion and vesicular transport; nuclear structure; RNA processing and modification; Transcription; Replication and recombination and repair were particularly elevated.

While to grow on the multimetal + lindane stress, the proteome related to carbohydrate transport and metabolism; lipid transport and metabolism; amino acid transport and metabolism; inorganic ion transport and metabolism; secondary metabolites biosynthesis and transport and catabolism; energy production and conversion; defence mechanisms; extracellular structures; posttranslational modification and protein turnover and chaperones; signal transduction mechanisms; translation and ribosomal structure and biogenesis were specifically depressed.

Remaining were proteins with general function (9%) or unknown functions (2%) which could contribute to intercept the challenges posed by multimetals and lindane stress.

**Fig. 3:**
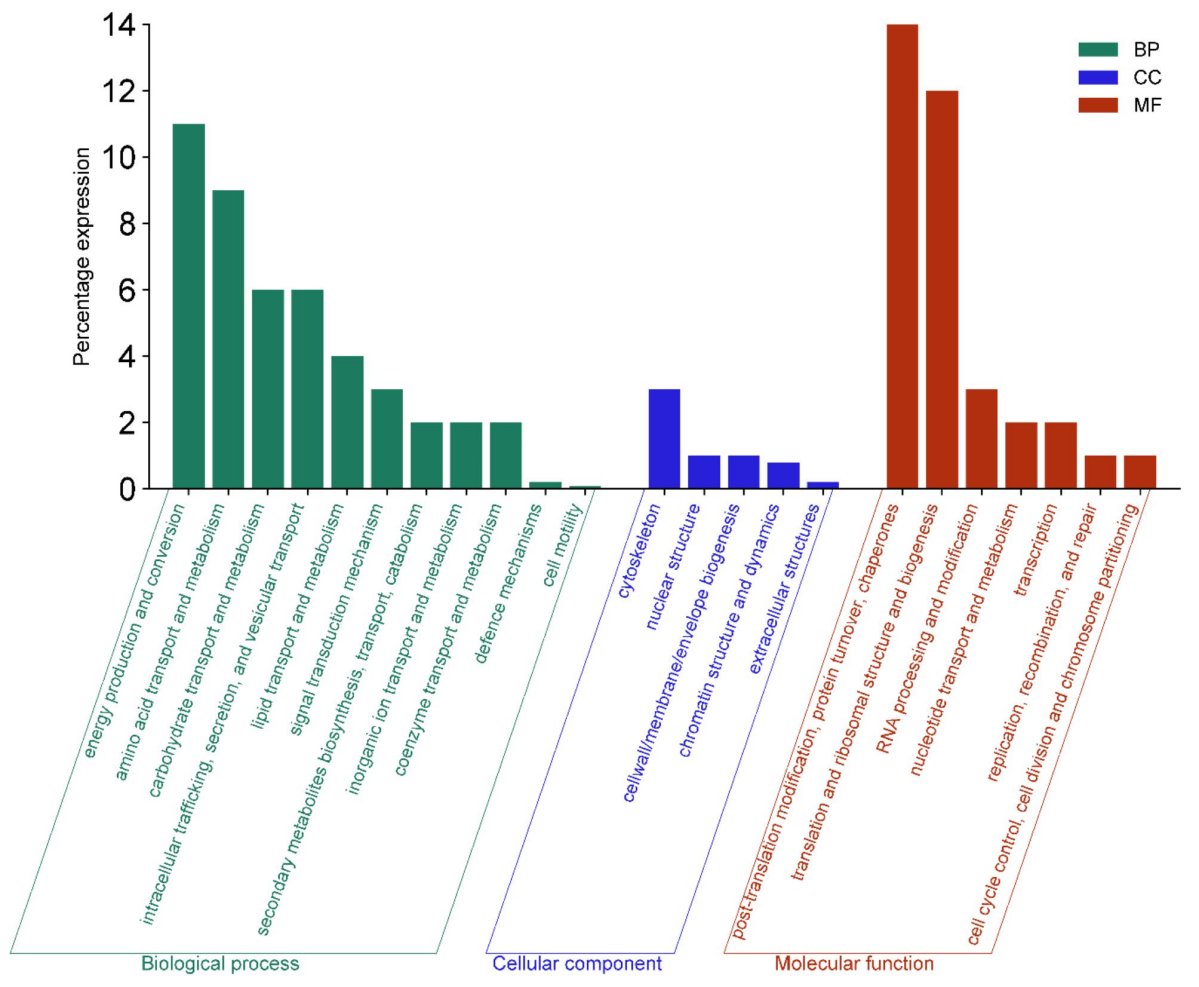
Percentage of proteome abundance classified according to biological process (BP), cellular component (CC), molecular function (MF) in *A. fumigatus* PD-18 in presence of 30 mg/L multimetal (MM) + 30 mg/L lindane exposure.

Figure 3 depicts the percentage of proteome abundance of the KOG classes. These proteins were linked to fungal physiology were broadly categorised under the functional groups of biological process, cellular component and molecular function^26^.

Biological Processes involves nutrient acquisition and metabolism; growth and development, including hyphal elongation and branching; reproduction both asexual (e.g., conidiation) and sexual; response to environmental stimuli, such as light, temperature, and pH.

Cellular Components includes formation of chitinous cell wall, plasma membrane containing ergosterol, cytoplasmic organelles, including mitochondria and nucleus and cytoskeleton composed of microtubules and actin filaments.

Molecular Functions include enzyme activity for metabolic processes; signalling pathways, including those involved in stress response and virulence; gene expression regulation, including epigenetic mechanisms; transport of molecules across membranes, such as nutrient uptake.

The highest percentage of proteome abundance was exhibited by post-translation modification and protein turnover and chaperones (14%) followed by translation and ribosomal structure and biogenesis (12%) belonging to subgroup of molecular function.

Table 1 depicts the fold changes in proteins of upregulated upto (3.0-fold) and downregulated upto (− 3.0-fold) with their locations inside the cell. With respect to the proteins’ location, proteins were majorly found at mitochondrial (25), cytoplasm (18), nuclear (14), extracellular (5), plasma membrane (4), peroxisome (1).

**Table 1:** Differential abundance and cellular location of highly regulated selected proteins in *A. fumigatus* grown in presence 30 mg/L MM + 30 mg/L lindane.

*p*-value	Protein accession number	Fold change	Protein KOG class	Protein name	Cellular location of protein
Upregulated					
0.001	Q4WX43	4.3	Transcription	ATP-dependent RNA helicase eIF4A	Nucleus
0.001	A0A084BH92	4.1	Secondary metabolites biosynthesis, transport and catabolism	Isoamyl alcohol oxidase	Cytoplasm
0.001	B0XQH1	4.1	Coenzyme transport and metabolism	60 S ribosomal protein L35	Nucleus
0.016	B0XZB6	3.9	Amino acid transport and metabolism	Mitochondrial enoyl reductase	Mitochondria
0.011	A4UTP0	3.9	Secondary metabolites biosynthesis, transport and catabolism	Carbonic anhydrase	Mitochondria
0.003	A2R0I8	3.8	Posttranslational modification, protein turnover, chaperones	26 S protease regulatory subunit 6B	Nucleus
0.013	Q4WM81	3.7	Posttranslational modification, protein turnover, chaperones	Serine/threonine-protein phosphatase	Cytoplasm
0.006	Q4WV94	3.7	Posttranslational modification, protein turnover, chaperones	D-amino acid oxidase	Peroxisome
0.0006	A0A084BM18	3.7	General function prediction only	Eukaryotic translation initiation factor 3	Nucleus
0.0003	Q4WLV1	3.6	Posttranslational modification, protein turnover, chaperones	Isochorismatase family hydrolase	Extracellular
0.005	A0A084C1K3	3.5	Translation, ribosomal structure and biogenesis	Glutamyl-tRNA synthetase	Nucleus
0.0004	B0XND8	3.5	Energy production and conversion	Endo-1,3-beta-glucanase Engl1	Extracellular
0.002	Q4WCZ1	3.5	Carbohydrate transport and metabolism	Acetylglutamate kinase	Mitochondria
0.0004	Q4WHV3	3.5	Secondary metabolites biosynthesis, transport and catabolism	GPR/FUN34 family protein	Plasma membrane
4.16141E−05	Q4 × 0E9	3.5	Amino acid transport and metabolism	NADH-ubiquinone oxidoreductase 49 kDa subunit	Mitochondria
0.0051	Q0CSN0	3.4	Energy production and conversion	Mitochondrial inner membrane translocase	Mitochondria
0.0002	A1CI51	3.4	Transcription	Kynurenine aminotransferase	Cytoplasm
0.0089	Q4WFD3	3.4	Intracellular trafficking, secretion, and vesicular transport	Coatomer subunit gamma	Cytoplasm
0.002	O60022	3.4	Transcription	RuvB-like helicase	Extracellular
0.0009	B0XXM2	3.4	Energy production and conversion	Class V chitinase	Extracellular
9.5897E−05	A0A084BQX5	3.3	Transcription	ATP dependent RNA helicase	Mitochondria
0.0003	A1C6S1	3.3	Amino acid transport and metabolism	ATP synthase oligomycin sensitivity conferral protein	Mitochondria
5.72245E−05	Q4WGP1	3.3	Carbohydrate transport and metabolism	Acetyltransferase component of pyruvate dehydrogenase complex	Mitochondria
0.0007	B0XVU3	3.3	Lipid transport and metabolism	Agmatinase	Extracellular
0.0009	Q4WUS8	3.2	Amino acid transport and metabolism	Phospho-2-dehydro-3-deoxyheptonate aldolase	Cytoplasm
0.002	A0A084BP78	3.2	Amino acid transport and metabolism	Acetolactate synthase	Mitochondria
0.010	Q4WXX9	3.2	Inorganic ion transport and metabolism	Pyruvate decarboxylase	Cytoplasm
0.003	A0A017SPG0	3.1	Signal transduction mechanisms	Calcium/calmodulin-dependent protein kinase	Cytoplasm
0.003	Q4WJ29	3.1	General function prediction only	Mitochondrial GTP/GDP transporter Ggc1	Mitochondria
0.0001	Q4WEJ7	3.1	Translation, ribosomal structure and biogenesis	Ubiquitin C-terminal hydrolase (HAUSP)	Nucleus
0.0004	P24634	3.0	Cytoskeleton	Tubulin alpha-2 chain	Cytoplasm
0.003	Q4WYW7	3.0	General function prediction only	Phosphatidyl synthase	Mitochondria
0.022	A0A084BPT4	3.0	Amino acid transport and metabolism	C1 tetrahydrofolate synthase	Cytoplasm
8.33938E-05	Q4WTW3	3.0	Cytoskeleton	Protein yop1	Plasma membrane
Downregulated					
0.021	A1CQY4	− 7.2	Translation, ribosomal structure and biogenesis	40 S ribosomal protein S26	Mitochondria
4.52448E−05	Q4WIF3	− 7.0	Inorganic ion transport and metabolism	Peptidyl-prolyl cis-trans isomerase	Nucleus
0.011	A1C821	− 6.8	Translation, ribosomal structure and biogenesis	60 S ribosomal protein L37a	Mitochondria
0.019	Q0CX81	− 6.5	Energy production and conversion	Plasma membrane ATPase	Plasma membrane
0.019	Q4WWC5	− 6.5	Posttranslational modification, protein turnover, chaperones	Histone H2B	Nucleus
0.001	A1CIN5	− 6.1	Secondary metabolites biosynthesis, transport and catabolism	Hydrophobin	Mitochondria
0.046	Q4 × 1E2	− 5.8	Posttranslational modification, protein turnover, chaperones	Ketoreductase	Cytoplasm
0.027	Q4WUF2	− 5.8	Translation, ribosomal structure and biogenesis	Ribotoxin	Nucleus
7.9874E−05	B8NAL4	− 5.7	Secondary metabolites biosynthesis, transport and catabolism	Glyceraldehyde-3-phosphate dehydrogenase	Cytoplasm
0.005	B0Y009	− 5.4	Posttranslational modification, protein turnover, chaperones	Phosphoglucomutase	Mitochondria
0.034	A0A084BEW6	− 4.9	Translation, ribosomal structure and biogenesis	60 S ribosomal protein L28	Mitochondria
3.43021E−05	A0A084BMR6	− 4.8	Secondary metabolites biosynthesis, transport and catabolism	Zinc-containing alcohol dehydrogenase	Mitochondria
0.032	B0XXL3	− 4.8	Carbohydrate transport and metabolism	Eukaryotic translation initiation factor 3	Nucleus
0.007	A1CPY9	− 4.7	Posttranslational modification, protein turnover, chaperones	Cell wall biogenesis protein phosphatase	Cytoplasm
0.028	Q4WTH0	− 4.4	Lipid transport and metabolism	Eukaryotic translation initiation factor 3 subunit F	Cytoplasm
0.043	Q5AVP8	− 4.2	Inorganic ion transport and metabolism	S-(hydroxymethyl)glutathione dehydrogenase	Cytoplasm
0.0005	B8NNR5	− 4.2	Inorganic ion transport and metabolism	Mitochondrial F1F0 ATP synthase subunit F (Atp17)	Mitochondria
0.003	A0A084BDZ8	− 4.1	Inorganic ion transport and metabolism	Catalase-peroxidase	Mitochondria
0.015	A1CPP3	− 3.9	Posttranslational modification, protein turnover, chaperones	Asp-hemolysin	Mitochondria
0.00007	Q0PIK5	− 3.8	Posttranslational modification, protein turnover, chaperones	Dolichyl-phosphate-mannose–protein mannosyltransferase	Plasma membrane
0.019588871	Q4WHE9	− 3.7	Carbohydrate transport and metabolism	Class V chitinase	Nucleus
3.06999E−05	A1CI95	− 3.7	Inorganic ion transport and metabolism	Catalase	Mitochondria
0.001588972	B0Y356	− 3.6	Posttranslational modification, protein turnover, chaperones	Small ribosomal subunit protein eS1	Nucleus
0.001640154	Q4WB06	− 3.6	Carbohydrate transport and metabolism	Alpha-1,4 glucan phosphorylase	Cytoplasm
0.000468742	B0XZ97	− 3.5	Energy production and conversion	Dihydrolipoyllysine-residue succinyltransferase	Mitochondria
0.045944264	I8A3C7	− 3.5	Cell cycle control, cell division, chromosome partitioning	Spermidine synthase	Nucleus
0.032749583	Q9Y713	− 3.3	Carbohydrate transport and metabolism	Beta-glucosidase	Cytoplasm
9.34438E−05	B0XYC7	− 3.3	Signal transduction mechanisms	Class II aldolase	Cytoplasm
0.001906189	B0XYA9	− 3.2	Energy production and conversion	Glucooligosaccharide oxidase	Mitochondria
0.027750948	Q0CFN1	− 3.1	Energy production and conversion	Serine protease	Mitochondria
0.049773045	Q0CZ22	− 3.1	Cell cycle control, cell division, chromosome partitioning	Coronin	Mitochondria
0.008940825	Q4WA07	− 3.1	Transcription	C2H2 finger domain protein	Nucleus
0.034378597	A1C5U3	− 3.0	Transcription	Methyltransferase	Cytoplasm

**Fig. 4:**
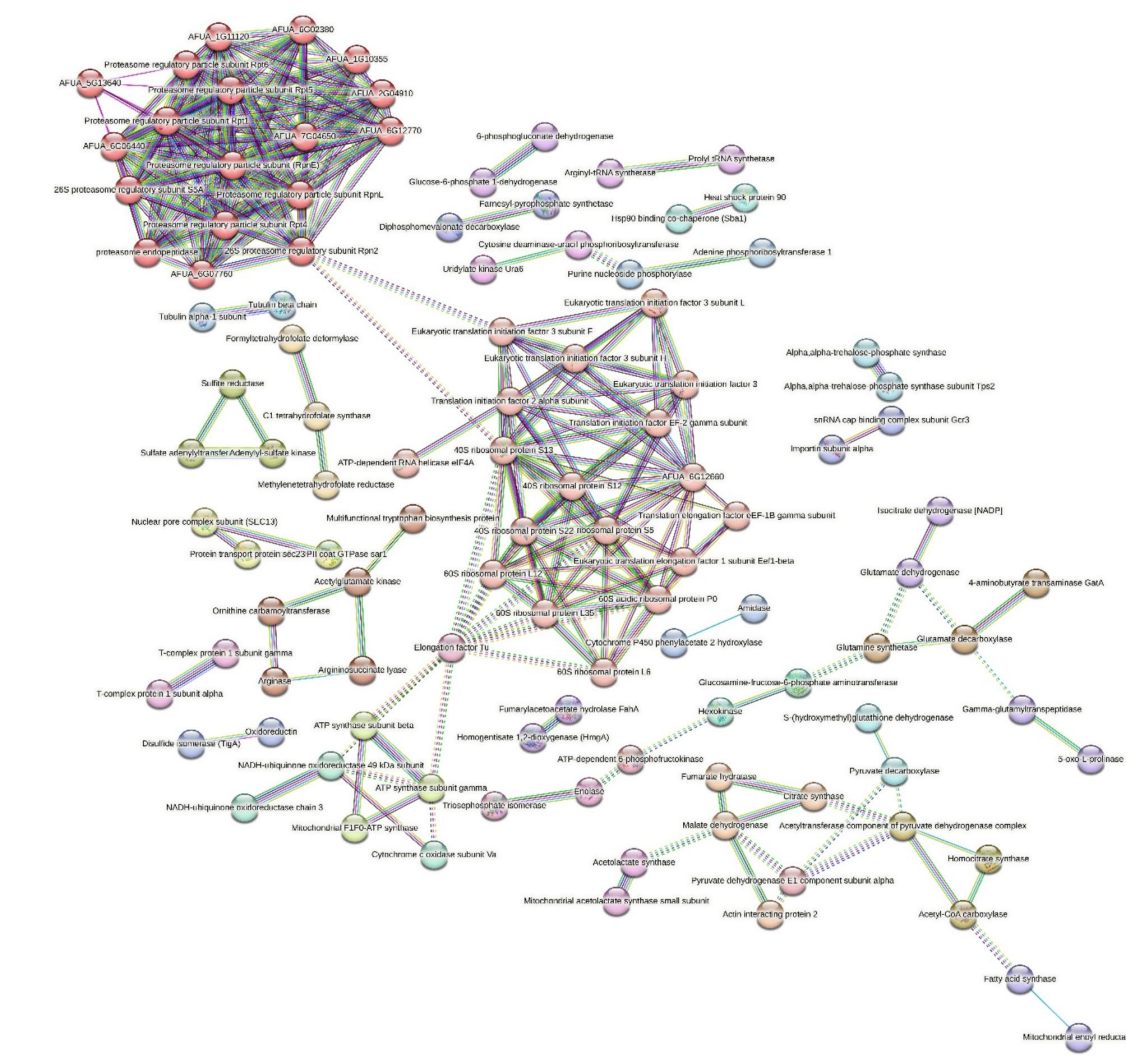
STRING analysis showing protein–protein interaction in *A.fumigatus* PD-18 in presence of 30 mg/L multimetal (MM) + 30 mg/L lindane. The colored lines depict the edges that show associations among various proteins including known interactions and predicted interactions (Light blue: proteins from curated databases. Pink: connect proteins that are experimentally determined. Dark green: connect proteins that occur as gene neighbourhood. Red: shows proteins with gene fusions. Dark blue: connect proteins with gene co-occurrence). There are 34 different clusters of proteins shown in various colors.

Figure 4 illustrates the interaction network generated by the STRING database for significantly upregulated proteins in *A. fumigatus* PD-18. The line thickness in the diagram corresponds to the strength of the associations. In this network, there are a total of 210 nodes, each representing a protein, and 295 edges, symbolizing associations. The average node degree stands at 2.81, and the average local clustering coefficient is 0.39.

Notably, the STRING analysis has identified 34 distinct MCL clusters of protein interactions. In this PPI network, cluster 1 comprised of proteosome complex and cross-presentation of soluble exogenous antigens (endosomes) with 18 protein counts followed by cluster 2 covering cytoplasmic translational initiation, ribosomal scanning and start codon recognition and Eucaryotic 48S preinitiation complex with 18 protein counts.

**Fig. 5:**
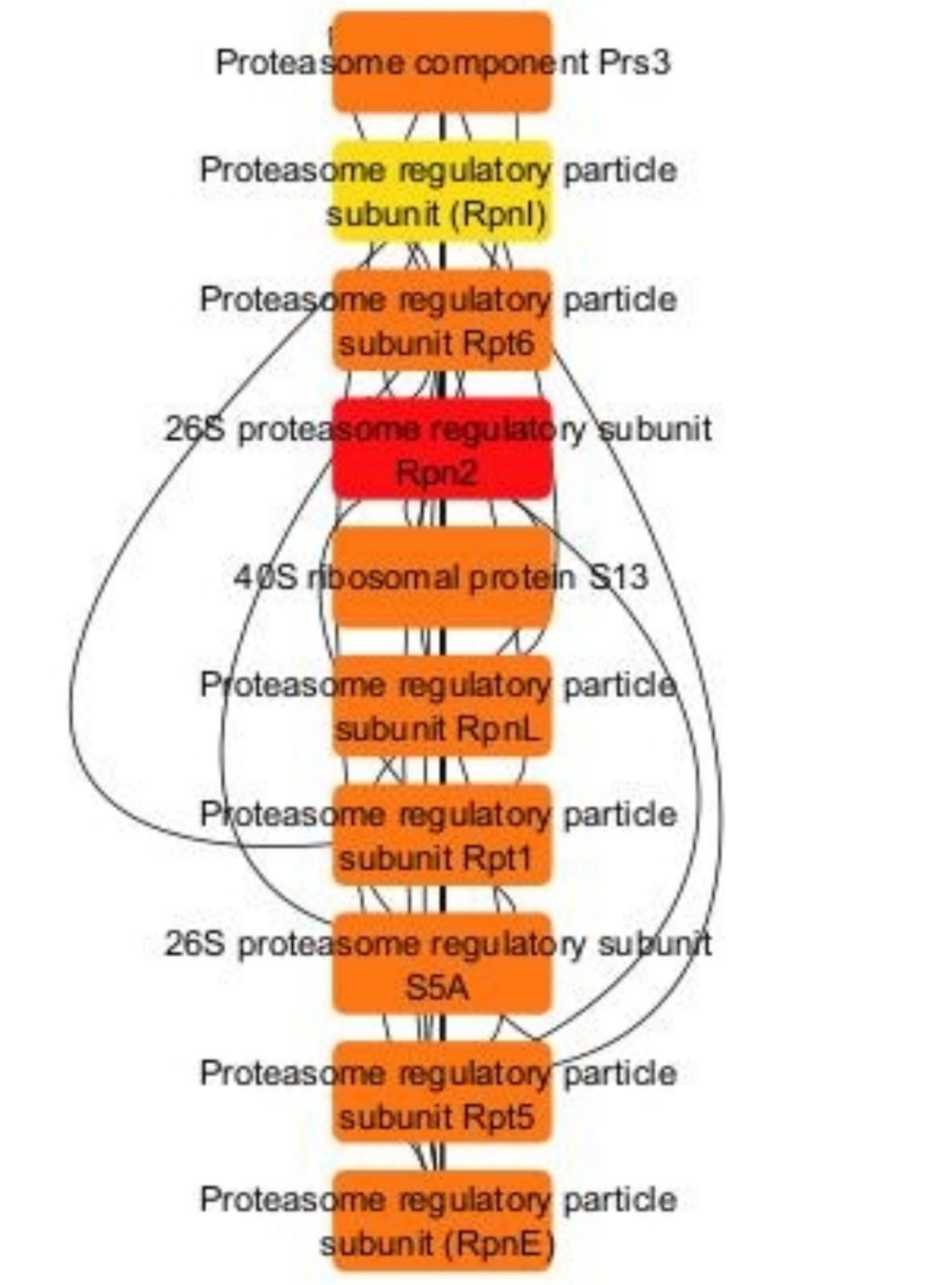
The first 10 proteins of the protein–protein interaction in *A.fumigatus* PD-18 in presence of 30 mg/L multimetal (MM) + 30 mg/L lindane was selected using CytoHubba plugin as red = high-ranking protein, yellow = low-ranking protein.

Figure 5 depicts the first 10 hub proteins of the protein–protein interaction selected using CytoHubba plugin. In protein protein interaction networks, hub proteins are important proteins that display more degree of interactions with other proteins.26 S proteasome regulatory subunit Rpn2 had the highest degree of interaction with 18 other proteins. While proteasome regulatory particle subunit (RpnI) had the least degree of interaction with 16 other proteins.

The proteins are arranged in descending order of their degree of interaction with other proteins and are as follows: 26 S proteasome regulatory subunit Rpn2 (18), Proteasome regulatory particle subunit Rpt5 (17), Proteasome component Prs3 (17), Proteasome regulatory particle subunit RpnL (17), 26 S proteasome regulatory subunit S5A (17), Proteasome regulatory particle subunit (RpnE) (17), Proteasome regulatory particle subunit Rpt1 (17), Proteasome regulatory particle subunit Rpt6 (17), 40 S ribosomal protein S13 (17), Proteasome regulatory particle subunit (RpnI) (16).

**Fig. 6:**
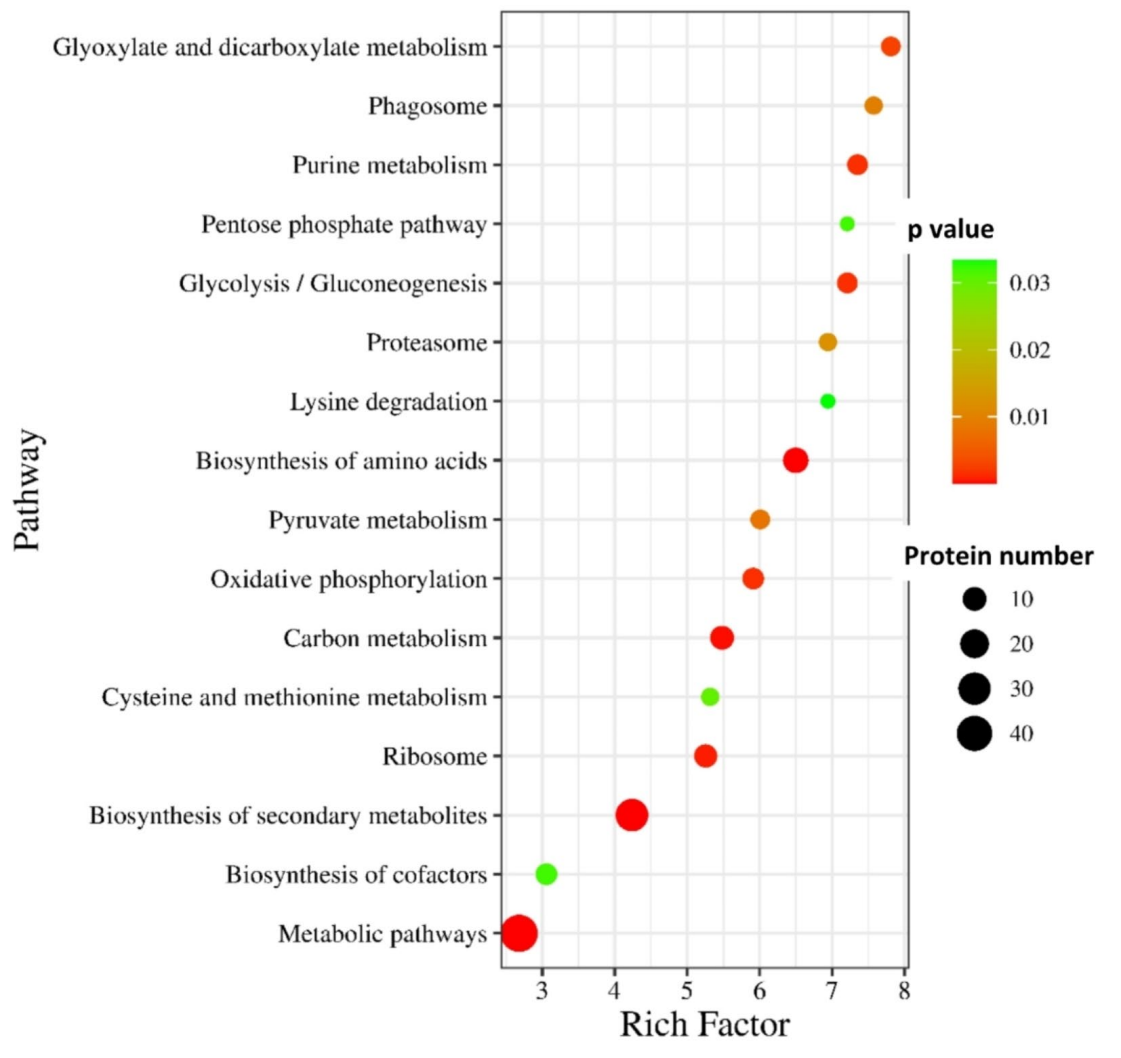
Pathway enrichment analysis of top 16 candidate proteins selected using ShinyGO in *A.fumigatus* PD18 in presence of 30 mg/L multimetal (MM) + 30 mg/L lindane. The red bubbles exhibit lower p-value and greater confidence on results than the green bubbles. Bigger size = more number of proteins.

Figure 6 depicts the KEGG pathway enrichment analysis that entails the rich factor which is the ratio of number of identified proteins in a particular category (e.g., those involved in mycoremediation) to the total number of proteins identified in the entire proteome. The highest enrichment factor corresponded to Glyoxylate and dicarboxylate metabolism which exhibits the maximum number of differential protein abundance of all the annotated proteins. The greater the rich factor, the greater the degree of pathway enrichment. The relatively small size of this bubble indicated ~ 10 proteins have been reported of this pathway out of which all exhibit differential protein abundance. While the lowest enrichment factor corresponded to Metabolic pathways with relative bigger bubble size indicating ~ 40 proteins. Lowest enrichment factor means highest number of annotated proteins. The top 3 differential protein abundance showing pathway with protein numbers are Metabolic pathways (~ 40), Biosynthesis of secondary metabolites (~ 30), Ribosome (~ 10). The top 2 highly enriched pathways are Glyoxylate and dicarboxylate metabolism and Purine metabolism significantly. The representative pathway as depicted by the biggest bubble and significantly abundant is Biosynthesis of secondary metabolites which is closer to mean highest richness factor of 10.

**Fig. 7:**
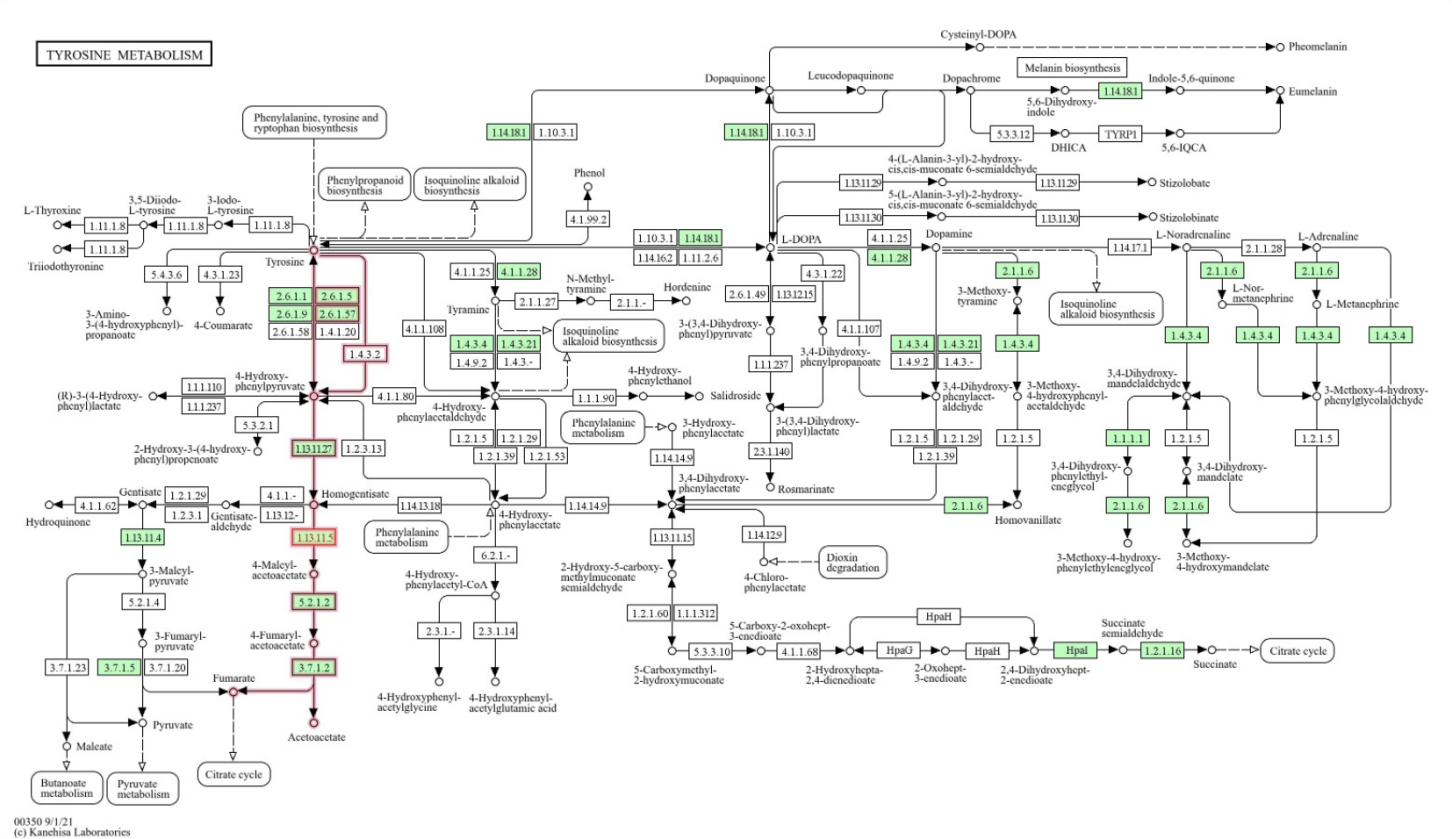
KEGG pathway modules of tyrosine metabolism of the enzyme homogentisate 1,2-dioxygenase (highlighted in red) in *A.fumigatus* PD18 in presence of 30 mg/L multimetal (MM) + 30 mg/L lindane.

Figure 7 depicts the KEGG pathway module of tyrosine metabolism. KEGG pathway modules reveal the occurrence of the enzyme of interest in any metabolic pathway. In a pathway, all the necessary reactions for the synthesis or metabolism of a specific product are displayed. A module represents a functional unit that is capable of being employed within a pathway, with certain modules being shared across multiple pathways. The enzyme homogentisate 1,2-dioxygenase is involved in the tyrosine metabolism which is related to lindane degradation through the involvement of enzymes and pathways that play a role in the breakdown of both tyrosine and lindane. Homogentisate 1,2-dioxygenase, catalyzes the conversion of homogentisate to 4-maleylacetoacetate followed by 4-fumarylacetoacetate and finally to acetoacetate and fumarate that culminates to citrate cycle.

## Discussion

The primary categories of proteins that play a pivotal role in conferring resistance and tolerance to *A. fumigatus* PD-18 against multimetals and lindane pesticide. These intrinsic and extrinsic proteins involved in metal assimilation and lindane degradation are expounded further.

### Mechanism of pesticide degradation by fungus

Fungi are equipped with diverse array of enzymes, metabolic pathways, and regulatory systems to break down pesticides. Pesticide degradation predominantly takes place extracellularly through intricate oxidative system that encompasses extracellular enzymes, redox mediators, and reactive oxygen species. Fungal bioremediation of pesticides occurs through various strategies like non-enzymatic bioaccumulation and enzymatic biotransformation and biodegradation. Bioaccumulation involves the accumulation of chemicals after rendering these molecules less toxic by demethylation, methylation, dehalogenation, hydroxylation, oxidation, and reduction methods. These molecules then chelate onto the fungal cells via biosorption or bioabsorption, followed by their subsequent dispersion within the internal organelles of the fungal biomass. Fungi primarily employ biotransformation and biodegradation as key mechanisms to detoxify or eliminate pesticides. The substances released into the environment through biotransformation typically undergo a change in molecular structure, often enhancing their solubility and reducing their biological activity by oxidation, reduction, hydrolysis, isomerization process. This alteration renders them more prone to degradation. The fungal enzymes exhibit wide-ranging substrate specificity, comprising of extracellular enzymes (laccases, manganese and lignin peroxidase), esterases, dehalogenases, intracellular enzymes (cytochrome P450 monooxygenases, dioxygenases, hydrolases) and may occur intracellularly/extracellularly (transferases, dehydrogenases)^19,27^. These enzymes facilitate the oxidation of various chlorinated pesticides, thereby exerting a substantial influence on their degradation process.

Majority of the degradative enzymes belong to the category of oxidoreductases of the six categories of enzymes (oxidoreductases, transferases, hydrolases, lyases, isomerases, ligases, and translocases) as per International Union of Pure and Applied Chemistry (IUPAC)^28^.

Hydrolases catalyse the hydrolysis of the substrate through the addition of water^29^.Enzymes like haloalkane dehalogenase produced by fungi facilitate the degradation of gamma-hexachlorocyclohexane (lindane) by catalysing the hydrolytic cleavage of carbon-chlorine bonds. This enzymatic action initiates the removal of chlorine atoms from lindane, transforming it into less chlorinated intermediates. As the chlorine is removed, these intermediates can undergo further enzymatic reactions, eventually leading to the formation of homogentisate^30^. This process is crucial for bioremediation, as it enables fungi to detoxify harmful organochlorine compounds like lindane, thereby reducing their environmental impact. In this study there was upregulation of the hydrolase enzyme haloalkane dehalogenase by 2.87-fold.

Oxidoreductases facilitate oxidation/reduction (redox) reactions by transferring electrons from a donor to an acceptor. Redox reactions are abundant in living cells and play crucial roles in essential biological processes like the tricarboxylic acid cycle, glycolysis, amino acid metabolism, and oxidative phosphorylation^29^. These fundamental enzymes are necessary for the cellular functions and vitality, being integral to the maintenance of cellular life and functionality during environmental stress. Short-chain dehydrogenase/reductase (SDR) enzymes are NAD- or NADP-dependent oxidoreductases in fungi that are vital for catalysing biochemical reactions and aid in the degradation of pesticide residues. In this study, there was upregulation (3.52-fold) of the enzyme oxidoreductase short chain dehydrogenase/reductase.

Amongst the twenty classes of oxidoreductases, important oxidoreductases include dehydrogenases that facilitate the transfer of hydrogen to an electron acceptor, oxygenases such as laccases, monooxygenases, dioxygenases utilize oxygen as the final electron acceptor, and peroxidases employ peroxides as the final electron acceptor in their enzymatic reactions. These non-specific oxidoreductases enzymes support the fungal growth on these xenobiotic compounds with arbitrary structures^18^. The catabolic strategy for degrading aromatic compounds involves two crucial steps: the activation of the stable benzene ring and its subsequent cleavage. In aerobic microbial degradation, oxygenases play a key role by activating the benzene ring through the incorporation of oxygen-containing substituents. The critical step of ring fission is then catalyzed by ring-cleaving dioxygenases, which break down the ring structure^31^.

In this study, there was downregulation of the peroxidase enzymes of cytochrome c peroxidase by (-2.2-fold) and heme peroxidase by (-1.83-fold). However, there was upregulation of the oxygenases enzymes of acireductone dioxygenase by (2.1-fold), homogentisate 1,2-dioxygenase by (1.7-fold).

The degradation of lindane by *A. fumigatus* involves a series of enzymatic reactions, with homogentisate 1,2-dioxygenase being one of the critical enzymes involved. This enzyme catalyzes the conversion of homogentisate, an aromatic intermediate metabolite, into 4-maleyl acetoacetate.

Transferases facilitate the transfer or exchange of specific groups (such as amino groups) between various compounds. This mechanism plays a crucial role in the production of necessary amino acids for protein synthesis in all cells during oxidative and environmental stress tolerance^29^. There was upregulation of aminotransferase family protein (3.93-fold); phosphoserine aminotransferase (4.09-fold); kynurenine aminotransferase (3.39); acetyltransferase (3.27-fold); 1,3-beta-glucanosyltransferase (2.04-fold), ATP phosphoribosyltransferase (1.5-fold).

Additionally, fungi mineralize the pesticide utilizing it as primary carbon and energy source as part of their metabolism. Fungi also cometabolize pesticides without utilizing it as direct carbon source by production of biosurfactants such as glycolipoproteins, sophorolipids, glycolipids that disperses the pesticide into small droplets and decreasing their surface tension and enhancing their bioavailability for fungal action^17,19,27^. Here we found upregulation of the protein glycolipid transfer protein by (3.54-fold).

### Mechanism of metal uptake by fungus

Fungi have the potential to endure high metal concentration and thus colonize metal contaminated environments^32,33^. Fungal metal detoxification process has evolved several metal resistance and tolerance stratagems. Extracellular mechanisms involve metal bioprecipitation, biosorption, biomineralization, biotransformation and bioleaching. While intracellular bioaccumulation is triggered by exposure to heavy metals that leads to the activation of multiple signalling pathways and metabolic processes aimed at preserving cellular homeostasis^34–36^.

Fungal resistance mechanisms thwart the metal uptake and its movement inside the fungal cell by regulating metal ion import. These approaches include passive absorption of metal ions by metal binding peptides and polysaccharides onto the cell wall components/cell surface^37^. Quantitative levels of enzymes such as β-1,3-glucanase, β-glucosidase, galactosidase, galactomanase, manase and their activity determine the saccharide and oligosaccharide biosynthesis for cell wall/cell membrane construction^38^. In this study there was (3.5-fold) upregulation in the enzyme endo-β-1,3-glucanase.The regulation of the enzymes are indicators of the extent of toxicity generated by heavy metals and pesticide contamination in the environment as observed by the downregulation of enzymes β-glucosidase by (− 3.3-fold) and α-galactosidase by (− 1.5-fold).

The chitinaceous cell wall of fungi have the ability to produce pigments such as cellular melanin^39^, extracellular polysaccharides e.g. pestan^40^, primary and secondary metabolites, amino acids, carboxylic acids, siderophores e.g. rhizoferrin^41^ that chelates heavy metals. Fungi are thus excellent biosorbents for a number of heavy metals which includes Ag, Ni, Cd, Cu, Pb, Zn^42^. Hence, these extracellular mechanism function within the cell to tackle heavy metals by evading the entry of these heavy metals and in the process the cell wall may get damaged. Class V chitinases belonging to the glycosyl hydrolase (GH) family participate in the breakdown of external chitin and responsible for utilization of fungal chitin as a source of energy during autolysis^43^. Here, we found the downregulation of the enzyme fungal/bacterial class V chitinase by (− 3.7-fold) and glycosyl hydrolase by (− 1.5-fold).

Formation of extracellular complexes and release of organic acid molecules such as oxalates, citrates, fumarate, succinate and other metal chelators that facilitate mobility of metals^44^. Oxalate synthesis in cells from glucose metabolism occurs by hydrolysis of oxaloacetate by oxaloacetase via glyoxylate oxidation^45^. Citrate is also an intermediate in the tricarboxylic acid cycle. The concentrations of heavy metals determine the synthesis of organic acids which supply the organic acid anion for metal-complexing and the protons forming insoluble precipitates of metal oxalate e.g. Cu oxalate^46^. Oxalate decarboxylase is involved in oxalate degradation and stabilization of the pH and concentration of oxalate anions in the external environment surrounding the fungal hyphae^47^. Here we found the upregulation of the enzymes citrate synthase by (1.41-fold) and enolase by (2-fold) which are involved in the tricarboxylic acid (TCA) cycle for generation of citrates. There was downregulation of the enzyme oxalate decarboxylase by (− 2.6-fold). Oxalates are the most potent chelator, as it creates complexes that are both insoluble and stable^48^. Thus, citrates and oxalates aided in heavy metal chelation.

Additionally, glycolipid transfer protein (GLTPs) are small, soluble proteins that accelerate the transfer of glycolipids between membranes^49^. GLTPs act as biosurfactants in fungi, significantly enhancing metal accumulation through several mechanisms. They facilitate the binding of metal ions to glycolipids, forming stable metal-glycolipid complexes that increase metal solubility and bioavailability. Additionally, GLTPs influence membrane lipid composition, enhancing the permeability for metal ions and helping regulate intracellular metal concentrations, which is crucial for maintaining metal homeostasis under stress conditions^50^. Thus, this process promotes efficient adsorption and absorption of metals, aiding in detoxification during bioremediation. Here we found upregulation of the protein glycolipid transfer protein by (3.54-fold).

Fungal tolerance mechanism towards excessive concentration of heavy metals largely involves intracellular heavy metal accumulation by localization and compartmentalization by polyphosphates inside the fungal subcellular organelle, vacuoles, cytoplasmic inclusions, cell wall by forming cation/polyphosphate complexes of mass between 1500 and 1700 Da through active transport process^51,52^.

Heavy metal sequestration and complexation by intracellular peptides i.e. metallothioneins (MTs) e.g. Cd, Cu, Zn metallothioneins^53^, phytochelatins (PCs), glutathiones (γ-Glu-Cys-Gly)^54^. MTs are thiolate sulfurs of cysteines, low molecular weight (< 10 kDa) proteins that are vital in shielding the fungal cell from the thiophilic metal ions such as Cd, Cu, and Zn^55^. Moreover, MTs have exceptionally high affinity for soft d^10^ metal ions such as essential Cu^2+^, Zn^2+^ and non-essential Cd^2+^, Hg^2+^ due to their large metal ion binding potential. The binding strength increases in the order of Zn^2+^< Cd^2+^< Hg^2+^ with increase in thiophilicity of metal ions due to their high thermodynamic stability^56^. In this study, there was no evidence of metallothioneins, or phytochelatins production, despite the presence of heavy metals Cd, Cu, Pb, and Zn. Typically, these metals are removed through glutathione-mediated sequestration, with GST expression depending on the type and concentration of heavy metal and treatment duration^57^.

However, glutathione and related peptides aid in regulating cadmium (Cd) levels in fungi. Specifically, the biosynthesis of these peptides, represented as (γ-EC)nG (where n ranges from 2 to 5), shows a preference for Cd, which may be due to the varying effects of different metals on metalloenzymes like transpeptidases, such as carboxypeptidase. These enzymes facilitate the formation of Cd-glutathione complexes, aiding in metal detoxification^58^. Glutathione functions as a crucial antioxidant and detoxifying agent, and enzymes like carboxypeptidase help regenerate it, ensuring a continuous supply for cellular protection. Additionally, the enzyme S-(hydroxymethyl) glutathione dehydrogenase is involved in metabolizing S-nitrosoglutathione (GSNO), a product of glutathione and nitric oxide (NO). By catalyzing the reduction of GSNO, this enzyme plays a vital role in detoxifying NO-induced damage, which is essential for the survival of fungi^29^. Here, we found the upregulation of the enzyme carboxypeptidase by (1.95-fold) and the downregulation of enzyme S-(hydroxymethyl) glutathione dehydrogenase by (0.7-fold) which are key players in the glutathione-dependent detoxification pathway. The toxic heavy metal ions sequester within the cell organelles of fungus, as the fungal cellular transporters fail to distinguish between the essential and nonessential heavy metal ions that have similar chemical properties^58^. Thus, the intracellular mechanism function to intercept the high concentration of metal toxicity inside the cell by detoxification process.

Further, heavy metals bring about oxidative harm to the cell membranes of fungi by production of reactive oxygen species (ROS) and stress response proteins (molecular chaperones, heat shock proteins). The ROS are detoxified by generation of antioxidants that are part of the thioredoxin system of NADPH dehydrogenases, superoxide dismutase, catalase, peroxidases that aids the fungus to encounter the reactive-oxygen species converting them into oxygen and water that amass inside the cell on metal exposure^59,60^. Chaperones primarily serve to prevent improper protein aggregation and facilitate the degradation of misfolded or damaged proteins. They transport metal ions to organelles and proteins that need metal for their functions. Heat shock proteins contribute to various processes including protein folding, transportation, maturation, and degradation^61,62^. Here, we found (3.2-fold) upregulation of Cu-Zn superoxide dismutase, coding for antioxidant protein and (1.6-fold) upregulation of heat shock protein Hsp98/Hsp104.

Further, presence of any form of stress intensifies the energy requirements in organism for survival. Likewise, when fungal cells encounter heavy metals and pesticide stress, they initiate the utilization of additional intracellular ATP reserves to maintain homeostasis^48^. F_1_F_0_-ATP synthases are enzyme complexes located in eubacterial plasma membranes, chloroplast thylakoid membranes, and the inner membranes of mitochondria that utilize the energy derived from a gradient of protons across the membrane to generate ATP from ADP and Pi^63^. Here, we found (1.5-fold) upregulation of cytochrome c oxidase, (1.2-fold) upregulation of ATP synthase proteins that are responsible for oxidative phosphorylation to produce energy for fungal cells and (1.6-fold) upregulation of mitochondrial F_1_F_0_-ATP synthase g subunit protein involved in transport of H^+^ ions responsible for metal chelation.

Additionally, *A. fumigatus* species is classified among thermo-acidophilic fungi^64^. This fungal genus possesses an internal pH control mechanism by actively expelling protons from the cell and maintaining low proton membrane permeability to facilitate cation assimilation. Further, the variations in the rate of biosorption of heavy metals into the fungal cell are associated with the functional groups present in the cell wall and its affinity for the heavy metals that in turn influences the number of receptors in the cell wall and membrane channels responsible for the uptake and release of heavy metals from the surrounding environment^13,38^.

Inositols play a vital role in the growth of numerous organisms including yeasts, fungi^65^.Among its various isoforms, myo-inositol holds a central position in cellular metabolism. The depletion of inositol in fungus leads to cell death^66^. There was (1.2-fold) upregulation of phosphatidylinositol transporter that has a role in osmoregulation in fungus. Myo-inositol forms unique structures with backbone of phosphate groups that are widely employed by eukaryotic cells to produce a diverse range of signalling molecules under stressful environment of micropollutants. Here, we found upregulation of myo-inositol-phosphate synthase by (2-fold) and downregulation of the enzyme inositol monophosphatase by (− 2.13-fold). Essentially, myo-inositol-phosphate synthase is involved in the initial synthesis of inositol compounds, whereas inositol monophosphatase plays a role in the breakdown of inositol phosphates by catalyzing their dephosphorylation^67,68^. This process leads to the production of free inositol which has significant role as versatile molecules that regulate various biological processes, including bioremediation.

Biological data, such as protein-protein interactions, gene regulations, cellular pathways, and signal transductions, can be effectively visualized and analyzed using network representations. By examining the network features of individual nodes, we can infer their significance within the network and identify crucial components that play central roles in biological networks^69–71^.

STRING network analysis (Fig. 4) reveals the proteasome regulatory particle subunits and the eukaryotic 48 S preinitiation complex of eukaryotic translation initiation factor 3 (eIF3) that play important roles in the protein-protein interaction network. The proteasome regulatory particle subunits, function as scaffolds and coordinate the activity and placement of multiple ubiquitin-processing factors at the proteasome. These large subunits act as central hubs in the protein-protein interaction network, interacting with various proteins involved in ubiquitination, protein unfolding, and translocation to the proteasome core. Thus, proteasome regulatory particle subunits are responsible for the recognition, deubiquitination, unfolding, and translocation of substrate proteins to the proteasome core for degradation or recycling for regulation of protein homeostasis in stressful environment^72^. The eIF3 complex, which includes the eIF3a subunit, is involved in the regulation of translation initiation in eukaryotic cells that participates in the formation of the preinitiation complex and prevents the premature binding of the 40 S and 60 S ribosomal subunits, thereby controlling protein synthesis. The eIF3a subunit can interact with and modulate the activation of signalling pathways, such as the ERK pathway, which is important for cellular processes like proliferation and differentiation^73^. Thus, proteasome regulatory particle subunits and the eukaryotic translation initiation factor 3 (eIF3) play a crucial role in the protein-protein interaction network of fungi by regulating translation initiation, modulating signalling pathways, and contributing to the stress response and adaptation under the combined exposure to multiple metals and lindane.

Hub proteins play a crucial role in predicting fungal responses to varying levels of contamination by integrating insights from protein-protein interaction (PPI) networks and functional analyses. These proteins, identified through network analyses, exhibit high connectivity, indicating their central roles in biological processes such as stress responses and detoxification mechanisms. By analyzing these networks, the proteins involved in the bioremediation of heavy metals and pesticide can be understood. Functional enrichment analyses, such as Gene Ontology and KEGG pathway analyses, reveal the biological processes associated with these hub proteins, enhancing understanding of how specific fungal species adapt to different contaminants which can serve as biomarkers for assessing the effectiveness of bioremediation. Biomarkers are biological indicators for assessing fungal remediation effectiveness, including enzymatic activity and metabolite production, which reflect the fungi’s ability to degrade pollutants and indicate successful detoxification.

CytoHubba revealed the 26 S proteasome regulatory subunit as a major cluster of hub proteins (Fig. 5) in the protein-protein interaction network. Fungi employ the 26 S proteasome regulatory subunit in bioremediation by selectively degrading damaged or misfolded proteins, which is vital for maintaining cellular homeostasis under stress, such as exposure to heavy metals and pesticides like lindane. This mechanism includes protein quality control through ubiquitin-tagged degradation, regulation of detoxification proteins to ensure only essential proteins are present, modulation of ligninolytic enzymes like laccases for breaking down complex pollutants, and adaptation to toxic environments by degrading inhibitors that hinder detoxification^74^. These functions enable fungi to thrive in contaminated settings and effectively perform bioremediation.

GO pathway enrichment analysis (Fig. 6) depicted that the differentially abundant proteins were significantly enriched within Glyoxylate and dicarboxylate metabolism and Purine metabolism as the major pathways. These pathways in fungi are integral for carbohydrate biosynthesis, nucleic acid production, energy homeostasis, signal transduction, and overall growth. The role of the Glyoxylate and dicarboxylate metabolism pathway is the biosynthesis of essential carbohydrates such as oxalates from fatty acids or two-carbon precursors, such as acetyl-coenzyme A. The crucial enzyme isocitrate lyase is involved in oxalate synthesis while the enzyme isocitrate dehydrogenase has a role in glutamate synthesis^75^. This was observed with the upregulation of isocitrate lyase enzyme by 2.4-fold and the enzyme isocitrate dehydrogenase by 2.0-fold.Purine metabolism is essential for the production of deoxyribonucleic acid (DNA) and ribonucleic acid (RNA) in eukaryotic cells. It provides the necessary building blocks for nucleic acid synthesis. Purine nucleotides derived from this pathway play crucial roles in maintaining cellular energy balance and signal transduction within the cell^76^.

KEGG tyrosine metabolism module (Fig. 7) revealed the pathway of the enzyme Homogentisate 1,2-dioxygenase that catalyzes by introducing molecular oxygen. This reaction is an important step in the further transformation and detoxification of lindane, ultimately leading to its complete mineralization. By catalyzing the conversion of homogentisate, this enzyme helps to channel the degradation pathway towards the production of less toxic metabolites, contributing to the overall detoxification of lindane by *A. fumigatus*.

In aerobic microbial degradation, aromatic compounds are typically converted into one of four key intermediates: catechol, protocatechuate, gentisate, or hydroquinone (benzene-1,4-diol). Additionally, related compounds, such as homoprotocatechuate, dihydroxyphenyl propionates, and homogentisate, can also serve as intermediates during the catabolic process^31^. Thus, the activity of homogentisate 1,2-dioxygenase is vital for the efficient degradation of lindane by fungus, corroborating the efficient mineralization (94%) of lindane by *A. fumigatus* PD-18^13^.

By analysing the GO pathway enrichment and KEGG metabolic pathway in addition to STRING protein–protein interaction network with the hub proteins, the underlying the adaptive strategies of signalling pathways that play a significant role in *A. fumigatus* PD-18 to cope with the cumulative stress of lindane and mixture of multiple metals were illuminated.

## Materials and methods

### Chemicals and reagents

Stock solutions of various heavy metals (10 g/L) were prepared by dissolving their respective salts, including Cd(NO_3_)_2_, Cu(NO_3_)_2_, K_2_Cr_2_O_7_, Ni(NO_3_)_2_, Pb(CH_3_COO)_2_, and Zn(NO_3_)_2_ in double-distilled water. A lindane stock solution (100 g/L) was created by dissolving lindane powder in HPLC grade acetone. These solutions were then adjusted to the required concentrations for our experiments. Deionized ultrapure water (RIONS Ultra 370 series) was used for preparing reagents and calibration standards, while all other chemicals utilized were of analytical grade and sourced from Merck, Sigma, and Qualigens.

### Microorganism and culture media composition

The fungal strain used in this study was *Aspergillus fumigatus* PD-18, which was originally isolated from the polluted banks of the Yamuna River in New Delhi, India. The genetic sequence of this strain was confirmed through 18 S rRNA gene sequencing and deposited in the Genbank (NCBI) database under an accession number KX365202^25^.

For fungal growth, composite medium was used, comprising the following components per liter: NH_4_NO_3_ (0.5 g), K_2_HPO_4_ (0.5 g), MgSO_4_.7H_2_O (0.1 g), NaCl (1.0 g), and yeast extract (2.5 g), with a pH maintained at 6.8 ± 0.2. The medium was sterilized by subjecting it to 121 °C for 15 min. Glucose (10.0 g/L) was introduced into the flasks separately after autoclaving to avoid precipitation.

### Methodology

In this investigation, we evaluated the alterations in protein abundance in *A. fumigatus* PD18 following exposure to a solution containing 30 mg/L of a combination of multiple metals (MM) and 30 mg/L of lindane. The multimetal solution comprised six distinct heavy metals - Cd, Total Cr, Cu, Ni, Pb, and Zn - each present at a concentration of 5 mg/L. Additionally, the growth media included 1% glucose. The selection of a 5 mg/L concentration for each heavy metal was based on the permissible limits for heavy metal content in irrigation water, as outlined by the Food and Agriculture Organization (FAO).

These prescribed limits for each heavy metal were as follows: Cd (0.01), Total Cr (0.1), Cu (0.2), Ni (0.2), Pb (5.0), and Zn (2.0). Furthermore, we considered the typical concentrations of heavy metals found in mixtures in the Yamuna River^77^. Pesticide lindane was selected as the co-contaminant as lindane occurs particularly in regions with high agricultural activity near the Yamuna River^78^. The biotic control samples consisted of composite media containing only 1% glucose.

To carry out our experiments, we utilized a set of 250 mL Erlenmeyer flasks filled with 100 mL of composite growth medium. Each flask was inoculated with one milliliter of spore suspension containing approximately 10^7^ spores, prepared by rinsing spores with sterile distilled water containing 0.01% Tween 80. These flasks were then placed in a 30 °C incubator with agitation at 150 rpm for 72 h to ensure the complete absorption of metal ions and degradation of lindane by the fungal culture. Following this incubation period, the flasks were retrieved, and fungal biomass was filtered. The analyses were performed in triplicate, separately for the biotic control and the samples treated with multi-metal and lindane.

### Proteomics analysis

#### Cell lysis and protein extraction

The fungal pellets were separated from the growth media by inverting and then centrifuging at 4000 g for 10 min at 4 °C. The mycelia were then rapidly frozen in liquid nitrogen, lyophilized, and stored as dry powder at − 20 °C for future use. To extract total protein from the dried fungal powder, a buffer containing 8 M Urea and 2 M Thiourea was used, following a modified protocol^79^.

The supernatant was obtained by centrifuging at 14,000 rpm and 4 °C for 10 min, followed by overnight precipitation in five volumes of ice-cold acetone. The resulting protein pellet was stored at − 20 °C for future use. To measure protein concentration, the Bradford assay was employed^80^. A stock solution of Bovine Serum Albumin (BSA) with a concentration of 2 mg/mL was prepared in water. A BSA standard curve was generated using concentrations ranging from 0 to 2.00 µg/µL. Coomassie Brilliant Blue dye reagent, prepared by mixing 95% ethanol and 100 mL of 85% (w/v) phosphoric acid, was added to the standard solutions. After a 5-minute incubation, the absorbance of the resulting solutions was measured at 595 nm.

#### Protein separation by 1D-SDS PAGE and in gel tryptic digestion of the proteins

Protein separation was achieved through one-dimensional SDS-PAGE, and the resulting protein bands were visualized using Coomassie brilliant blue staining. Each individual gel band was then excised into small sections and subjected to a series of treatments: washing with 10 mM ammonium bicarbonate, reduction with 10 mM 1,4-Dithiothreitol and 100 mM 2-Iodoacetamide, and tryptic digestion. The protein lysates were incubated overnight at 37 °C.

The resulting peptides were extracted from the gel pieces using 5 mM ammonium bicarbonate and subsequently purified using C-18 ZipTip columns, following the protocol^81^. Finally, the peptide lysates were dried using a vacuum centrifuge and stored at − 20 °C until they were ready for further analysis.

#### Mass spectrometric analysis

The dried peptide lysates were reconstituted using a solution of 0.1% (v/v) formic acid. Subsequently, they were loaded onto and separated by reversed-phase chromatography system for separation before undergoing measurement on mass spectrometer, specifically, the UPLC-coupled (Waters) LTQ Orbitrap Velos MS/MS (Thermo Fisher Scientific).

#### Data analysis (proteins identification and characterization)

The raw MS-MS ion spectra data acquired from the instrument were subjected to processing using Proteome Discoverer software (v1.4, Thermo Fisher Scientific)^82^. This processing considered both fixed and variable modifications, where carbamidomethylation at cysteines was considered a fixed modification, and oxidation of methionines as a variable modification. Only ranked one peptide hits with greater than equal to 1 of high confidence and < 1% false discovery rate (FDR) was accepted as identified and included in subsequent analyses.

The intensity data were then subjected to logarithmic transformation and normalization to determine changes in protein abundance, specifically focusing on those with at least a 1.5-fold increase or decrease in regulation. Significance was determined based on *p* < 0.05 being considered statistically significant. Triplicate gel runs were performed for both the biotic control and multi-metal and lindane treated samples.

#### Bioinformatic analysis

To functionally annotate the proteins, the generated lists of identified proteins were analyzed using prophane (https://www.prophane.de/). To create the heatmaps and volcano plots, we utilized the heatmap and volcano programs within the *R* tool version 3.2.0. For predicting the subcellular localization of regulated proteins, we employed the WoLFPSORT tool (https://wolfpsort.hgc.jp/).

#### Protein-protein interactions network construction and hub proteins analysis

Search Tool for the Retrieval of Interacting Proteins (STRING) database v12.0 (https://string-db.org/)^83^ was utilised to predict potential protein-protein interactions (PPI) among the upregulated proteins. A combined score of (> 0.9) (highest confidence score) was considered significant and degree of no more than 5 interactors was set as the cutoff criterion utilising Markov Cluster Algorithm for clustering. CytoHubba plugin of Cytoscape software v3.10.1 (https://cytoscape.org/)^69^ was used to analyse the important 10 nodes/hub proteins in the PPI network.

#### Functional network formation of protein abundance

Kyoto Encyclopaedia of Genes and Genomes (KEGG) (http://www.genome.ad.jp/kegg/)^71^ was used to identify the pathway module. ShinyGO v0.80 (http://bioinformatics.sdstate.edu/go/)^70^ was used to perform Gene Ontology (GO) and enrichment analysis to determine the functions of the overlapping protein abundance. The graphical display of the results was carried out using SRPLOT (https://www.bioinformatics.com.cn/en) to generate bubble dot.

## Conclusion

This study illuminates the molecular adaptations of *A. fumigatus* PD-18 to combat synergistic effects of lindane and multi-metal stress by producing a range of proteins that were either unique or upregulated compared to the control group. Majorly, the upregulated proteins belonged to the KOG category of posttranslational modification, protein turnover, chaperones. 26 S proteasome regulatory subunit was identified as lindane and multi-metals mixture stress biomarker in the fungus *A. fumigatus* PD-18. The most enriched pathways identified were Glyoxylate and dicarboxylate metabolism and Purine metabolism. Enzymes such as haloalkane dehalogenase and homogentisate 1,2-dioxygenase were revealed to be instrumental in lindane degradation in the presence of multiple metals. These new observations provide a crucial baseline profile of the *Aspergillus fumigatus* PD-18 of the genera *Ascomycetes* proteome. Further, this would serve as a suitable model for investigating the biotransformation and degradation of persistent inorganic and organic co-contaminants and streamline the design strategies for remediation of contaminated waste water systems.

## Electronic supplementary material

Below is the link to the electronic supplementary material.

Supplementary Material 1

## Data Availability

The data supporting the findings of this study are available from the corresponding author upon request. The mass spectrometry proteomics data have been deposited to the ProteomeXchange Consortium via the PRIDE [1] partner repository with the dataset identifier PXD031741.
