# Supplementary material for: Unveiling fungal strategies: Mycoremediation in multi-metal pesticide environment using proteomics
Source: Sci Rep. 2024 Oct 5;14:23171. doi: 10.1038/s41598-024-74517-y (PMC11457522; doi:10.1038/s41598-024-74517-y)
Supplement: Supplementary file 1 — Supplementary Material 1 [file 41598_2024_74517_MOESM1_ESM.docx]

**Supplementary figure**

**A**

**C**

**B**

**
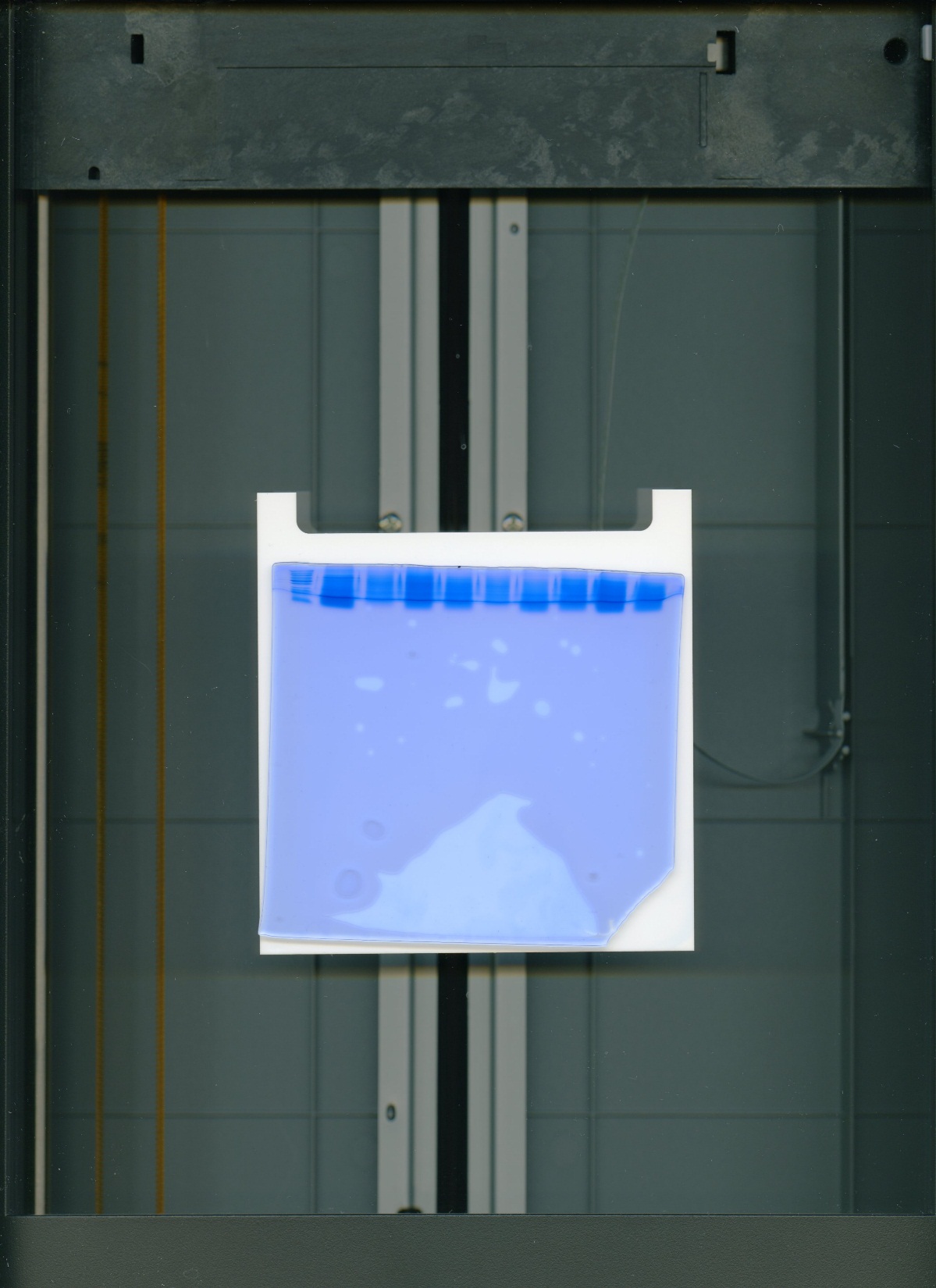
**

Supplementary figure: Segment of SDS-PAGE protein expression patterns in *A.fumigatus* (A) in absence of metal+lindane (Biotic control); (B) 30 mg/L Multimetal (C) 30 mg/L Multimetal+30 mg/L lindane
